# Differences in post-traumatic growth: Individual quarantine, COVID-19 duration and gender

**DOI:** 10.3389/fpsyg.2022.920386

**Published:** 2022-07-19

**Authors:** Keren Cohen-Louck

**Affiliations:** Department of Criminology, Ariel University, Ariel, Israel

**Keywords:** post-traumatic growth, gender, quarantine, COVID-19 duration, lockdowns

## Abstract

**Objective:**

This study focuses on positive effects of the COVID-19 pandemic and aims to identify associations between gender, individual quarantine and duration of the COVID-19 (short- medium- and long-term pandemic), and posttraumatic growth (PTG).

**Method:**

The data was collected via an online survey in Israel, and included 1,301 participants, 543 participants experienced short-term pandemics, 428 participants experienced medium-term pandemics and 330 participants experienced long-term pandemics. Most of the participants were female (73.6%), ranging from 18 to 89 years-old. The participants answered questions about their demographic background, individual quarantine experiences and ranked their PTG level.

**Results:**

The results indicate a significant main effect of gender and pandemic duration (short-, medium- and long-term pandemic). Women reported higher PTG levels than men, and participants experiencing short-term pandemic reported significantly lower PTG levels than participants experiencing medium- or long-term pandemic. There was also a significant interaction between gender and pandemic duration regarding PTG and a significant interaction in PTG by gender, pandemic duration and individual quarantine.

**Conclusion:**

The discussion addresses the findings in the context of traditional gender roles and gender differences in finding meaning and worth in home confinement situations.

## Introduction

The research on COVID-19 mainly emphasizes associations between the pandemic and negative psychological responses (Braun-Lewensohn et al., 2021; Levy and Cohen-Louck, 2021; López-Núñez et al., 2021). However, scholars have also identified positives outcomes of the COVID-19 pandemic and related lockdowns (Mancini and Mears, 2010; Chen et al., 2021; Levy and Cohen-Louck, 2021; Stallard et al., 2021; Cohen-Louck and Levy, in printing). COVID-19 hardships can lead to manifestations of mutual support and courage (Yu et al., 2021), and for some people, COVID-19 has facilitated positive behavioral and cognitive changes, such as spiritual growth (Stallard et al., 2021), greater appreciation of life, discovery and embracement of new possibilities, and has enables individual posttraumatic growth (PTG; Stallard et al., 2021). To identify factors that are associated with positive changes during the COVID-19 pandemic, this study focused on individual differences in PTG, a positive byproduct of physical or psychological traumas (Tedeschi and Calhoun, 1996; Stallard et al., 2021). PTG develops when people reevaluate their traumatic experiences by building new and positive life narratives (Tedeschi and Calhoun, 1996). PTG manifests in positive changes in self-perception, interpersonal relationships, and life philosophy (Tedeschi and Calhoun, 1996; Tedeschi et al., 2018). This can result in changes in five main areas: improvements in relating to others, greater personal strength, positive spiritual changes, a greater appreciation of life, and discovering and embracing new possibilities. Growth arises from the way the event is processed, not from the event itself. It leads individuals to recognize their vulnerabilities, what they can and cannot control, and compels them to reassess their personal priorities (Tedeschi and Calhoun, 1996). People who have experienced various types of trauma identify positive changes in their lives and improved mental health and wellbeing (e.g., Helgeson et al., 2006; Wu et al., 2019). Tedeschi and Calhoun (1996, 2004) consider PTG as a long-term outcome but also as a process that requires processing and elaboration of the traumatic event. This research explores the association between PTG, pandemic related characteristics and gender.

### Lockdowns and individual quarantine

In Israel, COVID-19 reached pandemic status on March 11, 2020 (Last, 2020). To contain COVID-19’s spread, the Israeli government issued a strict general lockdown on March 14, 2020. By mid-September 2020 a second lockdown was decreed and by January 8, 2021 a third lockdown was instated. Each lockdown lasted for about a month. During these lockdowns, Israeli citizens could leave their homes only for essential needs (e.g., buying groceries or medicines) and only essential personnel could go to work (Stein-Zamir et al., 2020). In addition to lockdowns, many countries established 14-day individual quarantines to isolate people who came from abroad (Rosca et al., 2020) as well as for sick or potentially infected individuals or those exposed to COVID-19 individuals. By the beginning of August 2021, the number of individual quarantines in Israel was close to 19.5 million (Data Gov, 2021).

Research on COVID-19 indicates that individual quarantines increase unemployment (Brooks et al., 2020), and the prevalence of posttraumatic stress disorder (PTSD), anxiety, depression, general distress, and fears associated with outdoor activities (e.g., Brooks et al., 2020; Lima et al., 2020). Thus, social isolation due to COVID-19 is related to adverse psychological effects (e.g., Gualano et al., 2020; Qiu et al., 2020). Unfortunately, studies tend to address the terms “individual quarantine” and “lockdown” interchangeably, even though they represent two significantly different situations. Lockdowns are for the general population and aim to increase social distancing to protect healthy people from becoming infected, whereas quarantines are on the individual, and aim to isolate an infected or potentially infected person. Moreover, individual quarantines represent a harsher state of isolation (Peak et al., 2020). For example, in Israel during the lockdowns, people could leave their homes for essential needs and outdoor exercise. However, during individual quarantines, isolated individuals are completely forbidden to leave their homes, and in some cases, even their room.

The research literature that specifically refers to individual quarantines mainly focused on medical aspects such as its effectiveness in preventing COVID-19 spread (Hossain et al., 2020; Shen et al., 2020). However, research on the psychological effects of individual quarantines, including PTG, is limited (Lau et al., 2006; Fekih-Romdhane et al., 2020). This study addresses this knowledge gap by identifying associations between experiences of individual quarantine and PTG. Individual quarantines represent a state of high social isolation (Peak et al., 2020) and are considered a type of adversity. Therefore, individual quarantines are associated with serious psychological consequences (Fekih-Romdhane et al., 2020; Fiorillo et al., 2020; Guessoum et al., 2020) including PTSD (e.g., Brooks et al., 2020), as well as personal growth and adaptive changes (Tedeschi and Calhoun, 2004; Shevlin et al., 2020). For example, during the SARS outbreak, many people who experienced isolation reported positive changes and growth (Lau et al., 2006). Considering these findings, that key aspects of individual quarantines (isolation and adversity) are associated with positive changes and growth, this study hypothesizes that:

H_1_: *There is a significant association between PTG and experiences of individual quarantine: Participants who experienced individual quarantines will report higher PTG than those who did not experience individual quarantines.*

### COVID-19 duration and posttraumatic growth

The majority of the studies on COVID-19’s psychological effects (e.g., Tull et al., 2020; Levy and Cohen-Louck, 2021) were conducted during the pandemic’s early stages. The COVID-19 pandemic has been around for more than two years, making COVID-19 a chronic, continuous threat. PTG is byproduct of adaptive processes that take time to emerge (Tedeschi and Calhoun, 2004). Therefore, to detect whether continuous COVID-19 leads to PTG, it is essential to compare between the effects of the short-term and prolonged experiences of the COVID-19 pandemic. The few studies that examined the effects of the COVID-19 duration on PTG levels (e.g., Feingold et al., 2022) addressed special populations such as health care workers (Feingold et al., 2022), therapists (Aafjes-van Doorn et al., 2021) and youth (Hyun et al., 2021). Those studies compared two waves of the pandemic and indicated that PTG increased from one wave to the next (Feingold et al., 2022). These findings are in line with the general research on continuous traumatic/stressors situations, indicating that the effects of exposure to ongoing mass traumas such as terrorism are positively associated with positive outcomes and PTG (Laufer and Solomon, 2006; Cohen-Louck and Saka, 2017). When coping with continuous uncertainty and existential threats, people tend to engage in psychological processes that facilitate a sense of coherence and understanding of these continuous adversities (Veronese et al., 2017). Considering that such psychological processes may manifest in PTG (Westphal and Bonanno, 2007), and based on the above studies, it is reasonable to hypothesize that:

H_2_: *There is a significant difference in PTG by the pandemic duration: Participants who experienced medium or long-term pandemics will report higher levels of PTG than participants who experienced a short-term pandemic.*

### Posttraumatic growth and gender

Research shows that gender is one of the key predictors of individual differences in responses to traumatic events in general (Fox et al., 2009; Shechory-Bitton and Cohen-Louck, 2020) and COVID-19 in particular (Ausin et al., 2020; Kirkman, 2020; Stevens, 2020). Compared to men, women exhibit higher levels of negative psychological effects (Kirkman, 2020; Platt, 2020; Stevens, 2020; Bonny-Noach et al., 2021; Levy et al., 2021), experience more sleeping problems, difficulties in staying at home during quarantines, feeling more worry and frustration Kirkman (2020); Platt (2020); Stevens (2020) and exhibit higher levels of PTG (Casali et al., 2021; Kalaitzaki, 2021). These findings are consistent with prior research, showing that women demonstrate higher levels of PTG than men in stressful and traumatic events (Jin et al., 2014). One of the explanations for this pattern suggests that women engage in reflection more than men (Prieto-Ursúa and Jódar, 2020). Reflection is critical to PTG development because it is associated with increased awareness of personal capacities and an awareness of the importance of social relations (Tolin and Foa, 2008). Additionally, women tend to use emotion-focused coping more than men (Konaszewski et al., 2021). Emotion-focused coping is an essential element in achieving a sense of acceptance following the aftermath of a traumatic event (Tedeschi and Calhoun, 2004). Since women are more inclined to use emotion-focused coping strategies, they are also more likely to achieve acceptance that is related to PTG (Jin et al., 2014).Therefore, the hypothesize states that:

H_3_: *There is a significant difference in PTG by gender: Women will report higher PTG levels than men.*

### The current research

This study aims to identify the association between PTG and gender, pandemic duration and exposure to individual quarantines. Most of the studies on the positive effects of COVID-19 were conducted following the first lockdown at the pandemic’s early stages, and knowledge on the impact of the pandemic’s duration is limited. The current research addresses this gap by comparing between individuals who experienced short-term (one lockdown), medium-term (two lockdowns) and long-term pandemic (three lockdowns). Additionally, although there are studies on the effects of quarantine (e.g., Brooks et al., 2020; Fernández et al., 2020), they mostly examine the impact of general lockdowns, whereas this study explores both the impact of general lockdowns as well as the effects of individual quarantines. Finally, this study explores the nature of gender differences in PTG. The findings of the current research will contribute to the understanding of COVID-19’s effects on PTG and the interrelationship between PTG, gender and pandemic-related factors.

## Method

### Participants

This study included 1301 participants from Israel. The majority were female (73.6%), and the age range was 18-89 years (*Mean* = 33.80, *S.D.* = 17.43). About half (48.2%) were secular, 23.5% traditional, and 28.3% religious. The majority (63%) were single, 30.8% married, 3.3% divorced and 2.9% widowers. The majority (72%) reported having an academic education, and the rest had a high school education. More than half of the participants (57.2%) experienced individual quarantine during the pandemic and 31 (2.4%) had COVID-19. The sample included three groups of participants in which 543 participants experienced short-term pandemics, 428 participants experienced medium-term pandemics and 330 participants experienced long-term pandemics (see Table 1 for demographic characteristics of each group).

**TABLE 1:** Associations between the demographic characteristics and pandemic duration.

	Pandemic Duration			*df*	χ*^2^*
	Short	Medium	Long		
**Gender**					
Female	71.5%	72.4%	78.8%	2	6.16*
Male	28.5%	27.6%	21.2%		
Total	100%	100%	100%		
**Religiosity**					
Secular	51.6%	49.9.%	44.1%	2	3.60
Religious	48.4%	50.1%	55.9%		
Total	100%	100%	100%		
**Family Status**					
Single	60.6%	75.2%	77.6%	2	37.25***
Married	39.4%	24.8%	22.45		
Total	100%	100%	100%		
**Employment**					
Unemployed	54.7%	55.6%	44.8%	2	10.50**
Employed	45.3%	44.4%	55.2%		
Total	100%	100%	100%		
**Education**					
High school	24.7%	35.3%	23.6%	2	17.37***
Academic	75.3%	64.7%	76.4%		
Total	100%	100%	100%		

*p < 0.05, **p < 0.01, ***p < 0.001.

### Measures

#### Background characteristics

The demographic questions addressed age, gender, religiosity, family status, educational level, and individual quarantines (yes/no).

#### Pandemic duration

The number of the lockdowns the participants experienced was defined as pandemic duration. The participants included three groups: 1) Short-term pandemic - individuals who participated during and shortly after the first lockdown (47%), 2) Medium-term pandemic - individuals who participated shortly after the second lockdown (32.9%), and 3) Long-term pandemic - individuals who participated shortly after the third lockdown (25.4%).

### Posttraumatic growth

To assess PTG, this study used the Hebrew version of the Posttraumatic Growth Inventory – PTGI (Tedeschi and Calhoun, 1996), which was validated by Laufer and Solomon (2006). PTGI assesses the positive changes following exposure to a traumatic experience and includes 21 items on a scale from 1 (no change) to 4 (significant change). The Cronbach alpha was 0.95.

### Procedure

#### Data collection

The current study is an online survey which was distributed by a link via social media outlets (Facebook, Twitter and WhatsApp). The data represents a snowball sample. The data was collected at three timepoints: 1) At the end of the first lockdown and following the first lockdown in Israel (1.4.2020-18.5.2020), 2) following the second lockdown (26.10.2020-17.11.2020), and 3) following the third lockdown (2.1.2021-7.2.2021). To ensure that the participants answer the questionnaires only once and that the samples are independent, the researcher took the following measures: 1. The link to the research was sent to potential participants only once and 2. The participants were instructed to fill the questionnaires only once. Furthermore, the participants were asked to state the last four digits of their ID number. Before the analyses, the researchers excluded from the data the few participants that answered the survey more than once (five participants were excluded from the second sample and seven from the third sample).

The study was approved by the ethics committee of the university (AU-SOC-KL-20200330). The questionnaire stated that: (1) participation in this study is anonymous, (2) the data will be used for research purposes only, (3) participants can withdraw from the study at any point, and they do not have to answer questions that makes them uncomfortable. All the participants gave their informed consent. It took about half an hour to answer the survey, and at the end, the participants were given information about helplines and support services. Participants who completed the survey answered all the question, therefore there was no missing data.

#### Data analysis

The data was analyzed using SPSS version 27. The general PTG score represents a mean score of all items. A chi-square test was initially used to examine the relationship between demographic variables and pandemic duration (short-, medium-, and long-term). To identify potential covariates, an ANOVA was conducted and PTG was included as a dependent variable, and family status, religiosity, and educational level were included as independent variables. Correlations between age, PTG and family status were also explored. Since there were no significant differences between single, widowers and divorced participants, family status was recoded into a dichotomic variable: 0 = not married, 1 = married. Secondly, an ANCOVA was conducted to examine the hypotheses about the differences in PTG by gender, COVID-19 duration and individual quarantine. Based on the exploratory results, family status, religiosity and employment were controlled for.

## Results

### Demographic characteristics by pandemic duration

The results of the chi-square test indicate a significant association between pandemic duration and gender, family status, employment and education (Table 1). The frequency of men among the participants who experienced short-term and medium-term pandemic was higher than the frequency of men among the participants who experienced long-term pandemic. The frequency of singles among the participants who experienced medium- and long-term pandemic was higher than the frequency of singles among the participants who experienced short-term pandemic. Unemployment was more frequent among participants who experienced short- and medium-term pandemic than among those who experienced long-term pandemic. As for education, high school level of education was more frequent among the participants who experienced medium-term pandemic than among the participants who experienced short-term or long-term pandemic. There was no significant association between pandemic duration and the participants’ religiosity.

### Descriptive findings: Posttraumatic growth

In general, participants reported medium levels of PTG (*Mean* = 2.63, *S.D.* = 0.98, *Range* = 1-4.95). The correlation between age and PTG was significant, but weak [*r* (1301) = −0.11, *p* = 0.001]. ANOVA indicates a significant main effect of religiosity [*F*(2, 939) = 11.59, *p* = 0.001, η*^2^* = 0.02]. Secular participants reported lower levels of PTG (*Mean* = 2.49, *S.D.* = 0.97) than traditional (*Mean* = 2.83, *S.D.* = 0.95) and religious participants (*Mean* = 2.76, *S.D.* = 1.02). According to Scheffe, there were no significant differences between traditional and religious participants. Additionally, ANOVA results indicate a significant effect of family status [*F*(4, 1296) = 6.34, *p* = 0.001, η*^2^* = 0.02] on PTG. However, since there were no significant differences between singles, divorced and widowed, family status was treated as a dichotomous variable (1 = married, 0 = unmarried) and a *t*-test was conducted. The t-test indicates (Table 2) significant differences by family status: PTG among married participants was lower than PTG among unmarried participants. As for employment, *t*-tests indicate significant differences by employment status: unemployed participants reported higher levels of PTG than those employed. There were no significant differences by educational level. Considering these associations between PTG and the demographic variables, family status, religiosity and employment were controlled for.

**TABLE 2:** Differences in PTG by family status, employment and education.

	PTG		Df	*T*	*Cohen’s d*
	Mean	S.D.			
**Family Status**					
Unmarried	2.71	0.95	713.97	4.82**	0.29
Married	2.42	1.00			
**Employment**					
Unemployed	2.73	0.95	1,299	3.91**	0.28
Employed	2.51	0.99			
**Education**					
High school	2.64	0.99	1,299	0.28	0.02
Academic	2.62	0.97			

*p < 0.05, **p < 0.01, ***p < 0.001.

### Posttraumatic growth, gender, pandemic duration, and individual quarantine

ANCOVA results indicate a significant main effect of gender [*F*(1, 927) = 5.15, *p* = 0.02, η*^2^* = 0.01] and pandemic duration [*F*(2, 927) = 3.76, *p* = 0.02, η*^2^* = 0.01] regarding PTG. There was no significant main effect of individual quarantine [*F*(1, 927) = 0.06, *p* = 0.80, η*^2^* = 0.00]. Women (*Mean* = 2.66, *S.E.* = 0.04) reported higher levels of PTG than men (*Mean* = 2.73, *S.E.* = 0.07). Participants who experienced short-term pandemic reported significantly lower levels of PTG (*Mean* = 2.41, *S.E.* = 0.08) than participants who experienced medium-term pandemic (*Mean* = 2.67, *S.E.* = 0.05) or long-term pandemic (*Mean* = 2.63, *S.E.* = 0.06). According to Scheffe, there was no significant difference between participants who experienced short-term and long-term pandemic.

**FIGURE 1:**
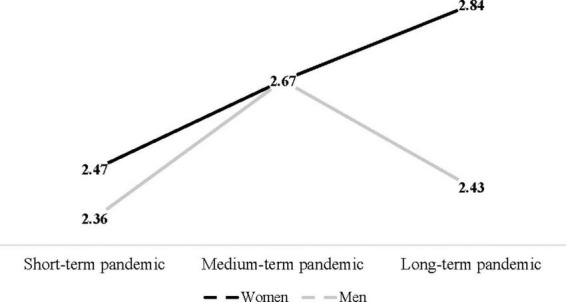
Interaction between gender and pandemic duration.

Additionally, ANCOVA (Figure 1) indicates a significant interaction between gender and pandemic duration [*F*(2, 927) = 3.37, *p* = 0.04, η*^2^* = 0.01]. Among women, PTG increased from the short-term pandemic (*Mean* = 2.47, *S.E.* = 0.09) to medium-term pandemic (*Mean* = 2.67, *S.E.* = 0.06), and from medium-term pandemic to long-term pandemic (*Mean* = 2.84, *S.E.* = 0.06). Whereas among men, PTG increased from short-term pandemic (*Mean* = 2.36, *S.E.* = 0.13) to medium-term pandemic (*Mean* = 2.67, *S.E.* = 0.09), but decreased in the long-term pandemic (*Mean* = 2.43, *S.E.* = 0.11).

**FIGURE 2:**
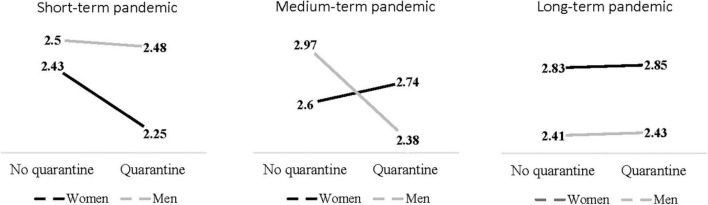
Interactions between gender, individual quarantine and pandemic duration regarding PTG.

The ANCOVA also indicates a significant PTG interaction by gender, pandemic duration and individual quarantine experience [*F*(2, 927) = 3.87, *p* = 0.02, η*^2^* = 0.01]. There was no significant difference by individual quarantine experience among women who went through short-term pandemic [F(1, 927) = 0.17, *p* = 0.91, η*^2^* = 0.00], medium-term [*F*(1, 927) = 1.51, *p* = 0.22, η*^2^* = 0.00] or long-term pandemic [*F*(1, 927) = 0.38, *p* = 0.89, η*^2^* = 0.00]. As for men, there was no significant difference by individual quarantine experience among those who went through short-term [*F*(1, 927) = 0.78, *p* = 0.38, η*^2^* = 0.00] or long-term pandemic [*F*(1, 927) = 0.01, *p* = 0.92, η*^2^* = 0.00]. However, among men who went through medium-term pandemic, there was a significant difference by individual quarantine experience [*F*(1, 927) = 11.4, *p* = 0.01, η*^2^* = 0.01]. Those who experienced individual quarantine reported lower PTG levels than those who did not experience individual quarantines (Figure 2).

## Discussion

This study identified associations between PTG and gender, experiences of individual quarantine and COVID-19 duration. One of the main findings indicates that PTG is associated with COVID-19 duration. Individuals who experienced medium- or long-term pandemic reported higher PTG levels than individuals who experienced only short-term pandemic. These findings partially correspond with our hypothesis (H_2_) and coincide with previous studies that found increases in PTG from one wave of COVID-19 to the second (Feingold et al., 2022). Considering that COVID-19 is a traumatic event (Horesh and Brown, 2020), the increase in PTG between short-term and medium or long-term pandemics supports the notion that PTG is associated with struggling through challenging events that shake the individual’s worldview (Tedeschi and Calhoun, 2004; Dekel et al., 2011). The struggle with a stressful event usually motivates constructive cognitive processing, leading to changes in perspectives about oneself, the event and the world (First et al., 2018) and may generate growth (Feingold et al., 2022). By relaxing more, adopting healthier lifestyles, and finding courage to overcome difficulties, people achieved personal growth during SARS and COVID-19 (e.g., Lau et al., 2006; Yu et al., 2021).

One of the intriguing findings that partially contradicts this study’s hypothesis is that individuals who experienced medium-term pandemic did not differ in PTG levels from those who experienced long-term pandemic. It is possible that the long-term pandemic did not increase stress, and therefore did not increase PTG. However, the interaction between pandemic duration and gender indicates a more complex pattern. In general, as hypothesized (H_3_), there was a significant gender difference in PTG, with women reporting higher PTG levels than men. These gender differences correspond with prior findings that women experience higher PTG than men (Jin et al., 2014). The interaction between gender and pandemic duration indicates that pandemic duration affects women and men differently. Thus, with each additional pandemic term, women reported higher PTG levels. Among men, there was an increase in PTG between the short-term and medium-term pandemic, but there was a decrease in PTG between medium-term and long-term pandemic.

Considering that pandemic duration increased the amount of time people spent at home with their families, this interaction suggests gender differences in the significance of home and family in the lives of men and women. Even with social changes and transformations in gender roles, women generally find more positive meanings in home and family activities and are more family- and home-oriented than men (Hughes and Kumari, 2017). Men still need to be more active outside their homes and tend to find more meaning in outdoor- and work-related activities (Levy et al., 2021). This finding corresponds with the findings that due to strong religious beliefs and military dominance, Israeli society is characterized by traditional gender values (Sasson-Levy, 2011; Mandel and Birgier, 2016; Yaish et al., 2021). Therefore, increases in home and family activities due to each additional pandemic term promoted PTG among women. Conversely, for men, the increase in family- and home-related activities promoted their PTG only during the early pandemic stages. Perhaps, men’s ability to find positive meanings in home- and family-related aspects of life is limited. The prolonged inability to experience outdoor- and work-related activities due to the long-term pandemic may have been more detrimental for men than for women, and manifested in a significant decrease in PTG among men.

As for the impact of individual quarantine on PTG, contrary to hypothesis (H_1_), there was no general difference in PTG by being exposed to an individual quarantine experience. Perhaps due to the multiple stress factors caused by COVID-19 (e.g., unemployment, lockdowns, fear of being infected), the individual quarantine’s impact on PTG was not salient enough. Nevertheless, the impact of an exposure to an individual quarantine experience can be salient when it coincides with additional factors such as gender and the pandemic duration. Therefore, there was a significant interaction between these variables. The individual quarantine experience had no significant effect on women’s PTG regardless of the pandemic duration. However, among men who experienced medium-term pandemic, individual quarantine was associated with decreased PTG. This pattern further supports our suggestions that men find more meaning in outdoor- and work-related activities, since factors that force men to stay at home (e.g., lockdowns, an individual quarantine) for relatively long periods are associated with decreased PTG. It is possible that the association between experiences of an individual quarantine and men’s PTG levels was evident only during the medium-term pandemic, because during the short-term pandemic there was not enough time to develop individual differences in PTG (Tedeschi and Calhoun, 2004; Helgeson et al., 2006), whereas by the long-term pandemic, men may have gone through a habituation process.

### Limitations and future research

This study is not without limitations. Firstly, due to the data’s cross-sectional nature, further research is necessary to detect causal pathways between PTG, pandemic duration and individual quarantine through a longitudinal design. Secondly, although this sample includes a relatively large number of participants, it is a convenience sample and therefore the external validity of this study is limited. Thirdly, the findings on gender gap in PTG may be related to the gender differences in emotional openness (Nellis, 2009). Additionally, to expand the understanding of the pandemic’s implications on PTG, future studies should examine the contribution of negative psychological symptoms. Finally, future studies should explore whether the cultural context and national patterns of coping with viral pandemic may affect PTG.

## Conclusion

This study expands the understanding regarding the psychological effects of individual quarantine and pandemic duration. Compared to women, the effect of the pandemic duration on men’s PTG was more complex and was associated not only with the pandemic duration, but also with their exposure to individual quarantines. The general pattern regarding men’s PTG indicates that factors increasing home confinement eventually led to diminished PTG. Considering that both pandemic duration and individual quarantine limit outdoor and work-related activities, this study’s findings suggest that women are more inclined than men to find meaning and worth in home- and family-related aspects of life. Theoretically, these findings imply the significance of traditional gender roles to the individual’s ability to achieve growth and development when facing continuous pandemic and individual quarantines. Clinically, the findings indicate men’s vulnerability to adverse effects of viral pandemics. Thus, clinical interventions and therapy during the COVID-19 and future pandemics should address men’s vulnerability and create gender specific interventions. Additionally, perhaps it will be beneficial to help men identify and find meaningful activities outside home when it is possible such as sport activities and trips or even to use virtual reality activities, which has possible public health benefits in intervention techniques that help alleviate the detrimental effects of prolonged lockdown periods (see Williams et al., 2020).

## Data Availability Statement

The raw data supporting the conclusions of this article will be made available by the authors, without undue reservation.

## Ethics statement

The studies involving human participants were reviewed and approved by Ariel University Ethics Committee. The patients/participants provided their written informed consent to participate in this study.

## Author contributions

KC-L contributed to this study’s conception, design, questionnaire development, organizing and analyzing of the database, writing and reviewing the manuscript, and approving the submitted version.
